# Measuring local depletion of terrestrial game vertebrates by central-place hunters in rural Amazonia

**DOI:** 10.1371/journal.pone.0186653

**Published:** 2017-10-17

**Authors:** Mark I. Abrahams, Carlos A. Peres, Hugo C. M. Costa

**Affiliations:** 1 School of Environmental Sciences, University of East Anglia, Norwich, United Kingdom; 2 PPG Ecologia e Conservação da Biodiversidade, Universidade Estadual de Santa Cruz, Ilhéus, Brazil; Università degli Studi di Napoli Federico II, ITALY

## Abstract

The degree to which terrestrial vertebrate populations are depleted in tropical forests occupied by human communities has been the subject of an intense polarising debate that has important conservation implications. Conservation ecologists and practitioners are divided over the extent to which community-based subsistence offtake is compatible with ecologically functional populations of tropical forest game species. To quantify depletion envelopes of forest vertebrates around human communities, we deployed a total of 383 camera trap stations and 78 quantitative interviews to survey the peri-community areas controlled by 60 semi-subsistence communities over a combined area of over 3.2 million hectares in the Médio Juruá and Uatumã regions of Central-Western Brazilian Amazonia. Our results largely conform with prior evidence that hunting large-bodied vertebrates reduces wildlife populations near settlements, such that they are only found at a distance to settlements where they are hunted less frequently. Camera trap data suggest that a select few harvest-sensitive species, including lowland tapir, are either repelled or depleted by human communities. Nocturnal and cathemeral species were detected relatively more frequently in disturbed areas close to communities, but individual species did not necessarily shift their activity patterns. Group biomass of all species was depressed in the wider neighbourhood of urban areas rather than communities. Interview data suggest that species traits, especially group size and body mass, mediate these relationships. Large-bodied, large-group-living species are detected farther from communities as reported by experienced informants. Long-established communities in our study regions have not “emptied” the surrounding forest. Low human population density and low hunting offtake due to abundant sources of alternative aquatic protein, suggest that these communities represent a best-case scenario for sustainable hunting of wildlife for food, thereby providing a conservative assessment of game depletion. Given this ‘best-case’ camera trap and interview-based evidence for hunting depletion, regions with higher human population densities, external trade in wildlife and limited access to alternative protein will likely exhibit more severe depletion.

## Introduction

Conservationists from across all major tropical and subtropical regions have voiced concerns that vast tracts of apparently intact forest mask large-scale faunal extirpation [1–11]. Where human communities extract tropical forest vertebrates, an “empty forest” scenario [12] may result, in which species larger than 2kg are virtually absent [13]. These defaunated forests may be subject to gradual degradation as the key functional roles played by megafauna are lost [14–16]. Although the importance of habitat fragmentation and degradation are recognised [17], hunting is often implicated as the main driver of defaunation [18] and heavily hunted sites have been shown to retain less than 20% of the crude vertebrate biomass of unhunted sites [5]. Biodemographic models predict that the adoption of firearms over traditional weapons results in depletion envelopes for low-fecundity, harvest-sensitive species, such as spider monkeys, around even low density, subsistence settlements in otherwise pristine remote forests [19]. Furthermore, the consumption by humans of hunted wild animals (bushmeat) has been implicated in the transmission of zoonotic diseases, with wide-ranging deleterious consequences for human health [20].

It is argued that subsistence hunters operate as optimal foragers rather than conservationists [21,22], almost always pursuing the most profitable prey irrespective of vulnerability. In multi-species prey assemblages, the persistence of harvest-insensitive species maintains the overall profitability of hunting, such that apparent competition drives vulnerable species to local extirpation [23,24]. Humans have been responsible for widespread faunal extinctions since prehistory [25,26]. Apparent cases of past stable coexistence with sensitive prey species may be an incidental consequence of low local human population density and inefficient hunting technology [27], and are irrelevant to modern conservation given the widespread adoption of firearms [28], increasing market integration [29] and human population growth even within protected areas [30].

Numerous measures of hunting sustainability have been proposed [31,32] and in a wealth of studies, the actual hunting offtake has been shown to be unsustainable for several species [33–35,9], resulting in areas of low prey biomass, low catch per unit effort, local extirpations and dramatically different prey offtake profiles [36–38]. In some regions, commercial hunting to supply urban demand (the “bushmeat trade”) is implicated as the key driver of overharvesting [39]. In other regions, however, even subsistence hunting practiced by isolated households can severely depress local populations of harvest-sensitive species [33]. Unsustainable hunting has been deemed an especially acute problem in tropical forests due to their global biodiversity importance [40], intrinsically low wild-meat productivity [41], high projected population growth [42], and often insufficient resources to enforce conservation regulations [43].

In stark opposition, some authors have argued that subsistence hunting, as practiced in indigenous, sustainable-use and extractive reserves, has little impact on populations of terrestrial game vertebrates [44]. Semi-subsistence and especially traditional communities often accumulate deep traditional ecological knowledge [45] and a well-developed conservation ethic stemming from both spiritual beliefs and a history of resource management [46–48]. The long period of coexistence between humans and vertebrate game [49] implies long-term hunting sustainability. Far from being motivated by short-term gains alone, subsistence hunters have complex culturally-mediated systems of resource utilisation rules and food taboos [50,51] including avoidance of vulnerable species, life-stages and seasons and the small and large-scale spatial rotation of hunting grounds [52,53], all of which enable faunal recovery.

Several studies have reported that despite long-term hunting offtake levels that consistently exceed predicted maximum sustainable yields, game depletion is not evident through changes in bushmeat availability at markets, catch per unit effort, prey profiles, per capita consumption rates or mean prey weights [54,55]. One explanation for this phenomenon is the replenishment through dispersal of hunted “sink” areas, by adjacent unhunted “source” areas [56]. This calls into question the aforementioned claims that game species are typically overharvested within intensively hunted portions of village catchment areas. Furthermore, it is claimed that the density estimates used as evidence of hunting depletion are flawed. Social or long-lived hunted species are able to change their behaviour in response to persistent hunting and other anthropogenic disturbance, such that they become less directly detectable [57–59]. Line-transect surveys therefore may fail to directly detect hunted species at hunted sites, whilst their presence can be confirmed by tracks and signs [60].

Even in cases where densities of game species are reliably found to be depressed in proximity to semi-subsistence communities, it is argued that this is insufficient evidence to substantiate unsustainable local hunting. Firstly, as Robinson and Redford (1994) [31] argue, depletion in itself does not entail a lack of sustainability. Offtake must by definition result in a spatio-temporally localised reduction in abundance and even where this depletion persists, yields may be maximised when a population is below carrying capacity. Secondly, environmental factors may be responsible for complex population changes [61]. Lastly, external and cryptic influences arising from the often conflicting use of tropical forest resources may be driving game depletion. Such influences include, (1) uncontrolled hunting practiced by illegal loggers, miners and commercial hunters from nearby urban areas [62] (2) a reduction in available wildlife source areas due to agricultural encroachment and forest fragmentation, and (3) increased pressure on terrestrial protein sources due to the overexploitation and pollution of marine and freshwater habitats.

These opposing views have important conservation implications. Some conservation biologists have argued for the need to prioritise the creation and enforcement of strictly protected areas that exclude humans and prevent hunting, in some of the world’s most biodiverse tropical rainforests [63–65]. Others have replied that the creation of large, strictly protected areas is (1) unethical, as they are either detrimental to the livelihoods of the world’s poorest people [66], or displace semi-subsistence communities entirely [67]; (2) unnecessary because both standing forest [68] and fauna can be effectively conserved by local communities [69]; and (3) counterproductive because (a) they damage relations with local communities who then become hostile to conservation [70] and (b) they remove the very people best placed to defend biodiversity [71,44], as local communities are relatively permanent and cost-effective deterrents of commercially motivated external agents of environmental degradation such as commercial hunters, logging companies and large-scale cattle ranchers, who have little incentive to conserve wildlife. Cognisant of these arguments, most conservation practitioners advocate a mix of strict and multi-use protected areas that acknowledge the use-rights of local communities. Here we aim to contribute to this debate by assessing the degree of depletion of a range of neotropical forest vertebrates in the vicinities of semi-subsistence communities and towns in Brazilian Amazonia using both camera trapping and interview surveys. We do not necessarily restrict the term “depletion” to demographic reduction via hunting offtake, though we consider this an important mechanism. We therefore use the term “depletion” for either depletion or repulsion. Equally, assessing the long-term sustainability of hunting in these regions is beyond the scope of this study. Instead, we provide a snapshot of the status of forest vertebrate populations.

We hypothesise that (1) Harvest-sensitive species, including large-bodied species such as tapir, highly preferred game species such as white-lipped peccary and low-lambda species such as Ateline primates, are depleted in proximity to semi subsistence communities in our study regions. This depletion will be evident through both (a) lower camera trap detection rates in proximity to communities, especially large communities and (b) longer interview-reported distances from communities to encounter sites of any given species; (2) Fast-reproducing (high-lambda), disturbance-tolerant species such as agoutis, which benefit from opportunities to raid agricultural plots, will be relatively more abundant in proximity to communities; (3) Nocturnal and cathemeral species, whose circadian activity patterns permit minimizing direct contact with humans, will be relatively more common in proximity to communities; (4) Cathemeral species, including brocket-deer and felids, will alter their behaviour in order to reduce human encounters, thereby exhibiting higher detection rates at night when in proximity to communities; (5) The cumulative detected biomass of terrestrial vertebrates will be significantly depressed in proximity to communities; and (6) Overall detection rates will be lower in lower productivity black-water river basins, where the relative impact of hunting will be more severe.

To this end we make several key assumptions. Firstly, we assume that proximity to human settlements is a proxy for the intensity of hunting and other anthropogenic disturbance. This rests on the well supported observation that hunters behave as central place foragers [72], such that hunting intensity declines from the centre of the community. Secondly, we assume that areas near human settlements are not otherwise intrinsically hostile to forest vertebrate species. To the contrary, it is anticipated that human settlements were deliberately established in environmentally favourable high-productivity locations. For example, we expect a higher human population density in areas allowing greater access to abundant natural resources as predicted by an ideal-free distribution. These resources include a higher soil fertility which mediates the density and species richness of nonvolant mammals across Amazonia [73,74]. Lastly, we assume that commercial hunting in our study regions represents a negligible fraction of the total offtake, which we are not “missing” by surveying subsistence hunting at the scale of local communities. This is plausible because (1) our study regions do not contain large urban populations and (2) culturally, hunted meat is not much sought by local urbanites (Projeto Médio Juruá, unpublished data).

## Methods

All research involving human participants was approved by the University of East Anglia Ethical Review Board. Informed consent was obtained from all interview participants.

### Study area

**Fig 1:**
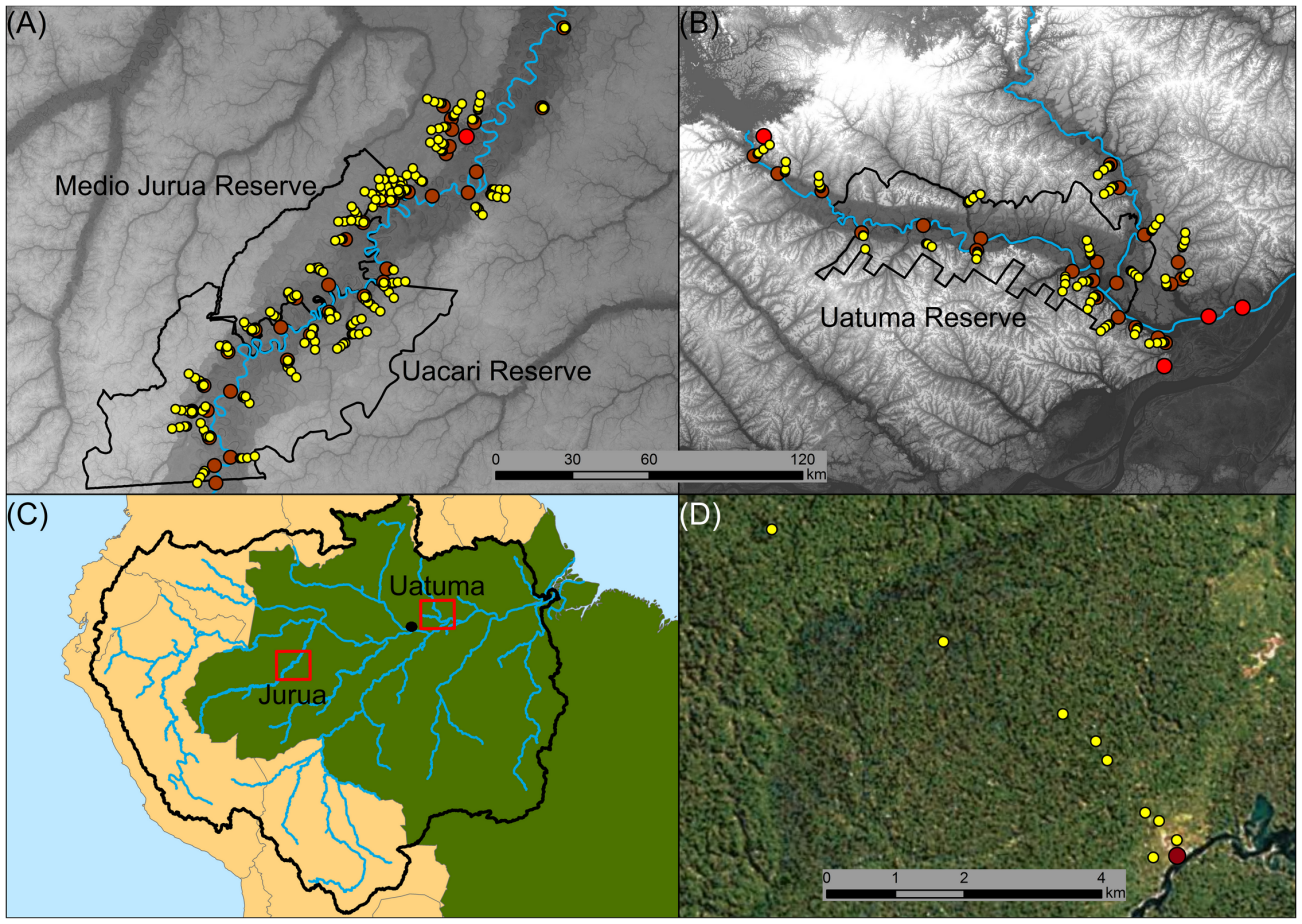
Geographic location of Juruá and Uatumã study regions within the Amazon basin, and the distribution of camera trap stations at an example local community. (A) Juruá study region. (B) Uatumã study region. (C) The Amazon basin. (D) The distribution of camera trap stations at an example local community. Major rivers are represented as blue lines, towns as red circles, communities as brown circles and camera trap stations as yellow circles. Background in panels A and B, which are presented at the same scale, display elevation, with darker shades indicating lower elevation. Sustainable use and extractive reserves are outlined in black. The Amazon basin (panel C) is outlined in black, the two study areas are outlined in red and Manaus is represented by a black circle. In panel D, the four camera trap stations nearest one of the sampled communities were placed within the peri-community agricultural mosaic, whilst five other stations were within contiguous primary forest.

This study was carried out in the Médio Juruá and Uatumã regions of Western and Central Brazilian Amazonia (Fig 1). The Médio Juruá study region covers an area of 1,637,008 ha and consists of 63.9% of primary unflooded (*terra firme*) forest, 30.0% of seasonally-flooded *várzea* forest, 4.4% of permanent water bodies, which include the Juruá River (the second-largest white-water tributary of the Amazon) and its tributaries and oxbow lakes, 1.8% deforestation and 0.1% natural non-forest. Two sustainable-use reserve—the Uacari Sustainable Development Reserve and the Médio Juruá Extractive Reserve—jointly protect 42.3% of this landscape. The nearest towns are Carauari, which is 88 fluvial km downstream from the Médio Juruá Reserve and has a population of 4145 families (~23500 people), and Itamarati, which is 120 fluvial km upstream from the Uacari Reserve and has a population of 905 families (~5130 people).

The Uatumã study region covers an area of 1,601,704 ha comprised of 62.3% of undulating upland primary unflooded (*terra firme*) forest, 17.9% of primary low-lying and seasonally-flooded *igapó* forest, 11.1% of permanent water bodies, which include the Uatumã River (which connects the Balbina reservoir to the Solimões River) and its main tributary the Jatapú River, 4.0% deforestation and 4.7% natural non-forest, which includes areas of *campina* and *campinarana* non-forest vegetation on oligotrophic sandy soils. The Uatumã Sustainable Development Reserve legally protects 27.0% of this landscape. The nearest towns are Vila Balbina, which has a population of 420 families (~2380 people) and is 66 fluvial km upstream of the reserve, and São Sebastião, Itapiranga and Urucará, with populations of 1214, 1345 and 2051 families, respectively, and are 37, 40, and 53 km fluvial distance downstream of the reserve, respectively.

Both regions are inhabited by *ribeirinhos* who are former rubber-tapper semi-subsistence communities of mixed-descent, with producer cooperatives and resource-management programs. Communities in both regions practice terrestrial subsistence hunting using firearms, typically 16- and 24-gauge shotguns. Terrestrial hunting with bows, snares and shotgun traps was negligible, Large-scale ecological and socioeconomic differences between the two study regions are due to drainage geochemistry and proximity to Manaus, the largest city (~2.05 million people) in the state of Amazonas. The Juruá region encompasses highly productive white-water floodplain ecosystems, whereas the Uatumã region encompasses much less productive black-water ecosystems, potentially resulting in lower faunal biomass density at Uatumã. Secondly, the Juruá region is over five times farther from Manaus, which increases transaction costs and reduces market opportunities for Juruá inhabitants.

### Vertebrate species

For all species that were either the subject of interviews or reliably detected by camera traps, a series of species traits were compiled (Table 1). Species taxonomic relatedness was also assessed as a proxy of phylogenetic similarity (S1 File, Appendix A). Values for species intrinsic rate of population increase (lambda) were taken from Robinson and Redford (1991) [75] and C.A. Peres (unpubl. data). Values for adult body mass per species was taken from Dunning, 1992, Carboneras, 1992, Baptista *et al*., 1997, Emmons and Feer, 1997, Nowak, 1999 and C.A. Peres (unpubl. data) [76–80], with the mean of male and female adult body mass used for sexually dimorphic species. Where only a range of adult biomass was known for a given species, the median of the upper and lower limits was used. Values for mean group size per species were taken from the Projeto Médio Juruá faunal monitoring program based on 90 transects of up to 5km in length censused on a monthly basis for up to 34 months [81]. Species were assigned a categorical activity pattern [diurnal, nocturnal or cathemeral], based on the camera trap detections (S1 File, Appendix B). Species were assigned a score reflecting their propensity to enter agricultural plots, as reported by semi-subsistence agriculturalists in the Juruá region [82] (see Local Interviews below and S1 File, Appendix C). Lastly, species were assigned a region-specific score reflecting the frequency with which they are hunted at each study landscape, as reported by respondents in both the Juruá and Uatumã regions.

**Table 1:** Study species reliably detected by camera traps or the subject of interviews and their key traits.

**Species binomial**	**English name**	**Species code**	**Dataset**	**Lambda (λ)**	**Body mass (g)**	**Group size**	**Hunt Juruá**	**Hunt Uatumã**	**Activity pattern**
**Mammals**									
*Mazama americana*	Red Brocket Deer	Maza.am	both	1.42	30000	1.1	8.3	4.8	cathemeral
*Mazama nemorivaga*	Grey Brocket Deer	Maza.ne	both	1.61	18000	1.2	1.3	2.9	cathemeral
*Pecari tajacu*	Collared Peccary	Peca.ta	both	2.01	25000	4.9	16.5	15	diurnal
*Tayassu pecari*	White Lipped Peccary	Taya.pe	both	1.58	32000	68.3	21.1	1.9	cathemeral
*Atelocynus microtis*	Short Eared Dog	Atel.mi	camera	1.15	7750	1.2	0	0	diurnal
*Leopardus pardalis*	Ocelot	Leop.pa	camera	1.58	15000	1.3	0	0	cathemeral
*Leopardus wiedii*	Margay	Leop.wi	camera	1.58	6000	1	0	0	cathemeral
*Panthera onca*	Jaguar	Pant.on	both	1.26	80000	1.4	0	0	cathemeral
*Puma concolor*	Puma	Puma.co	both	1.36	45000	1.1	0	0	cathemeral
*Puma yagouaroundi*	Jaguarundi	Puma.ya	camera	1.58	8000	1	0	0	diurnal
*Eira barbara*	Tayra	Eira.ba	camera	1.32	4850	1.3	0	0	diurnal
*Nasua nasua*	South American Coati	Nasu.na	camera	1.26	5100	11.9	0	0	diurnal
*Procyon cancrivorus*	Crab-Eating Raccoon	Proc.ca	camera	1.39	5400	1	0	0	nocturnal
*Priodontes maximus*	Giant Armadillo	Prio.ma	both	1.8	6000	1.2	0	0	nocturnal
*Nonspecific Cingulata small*	Small Armadillo	Nons.Ci	camera	1.905	30000	1	2.8	8.1	nocturnal
*Didelphis marsupialis*	Common Opossum	Dide.ma	camera	5	1087.5	1	0	0	nocturnal
*Metachirus spp*	Four-Eyed Opossum	Meta.sp	camera	5.2	390	1	0	0	nocturnal
*Tapirus terrestris*	Brazilian Tapir	Tapi.te	both	1.22	160000	1.2	5.4	5.3	nocturnal
*Myrmecophaga tridactyla*	Giant Anteater	Myrm.tr	both	1.7	30500	1.2	0	0	diurnal
*Tamandua tetradactyla*	Southern Tamandua	Tama.te	camera	1.62	4500	1.1	0	0	nocturnal
*Alouatta spp*	Howler Monkey	Alou.sp	interview	1.17	6500	6.2	4.1	0	diurnal
*Ateles spp*	Spider Monkey	Atel.sp	interview	1.08	9020	11.7	0	0	diurnal
**Species binomial**	**English name**	**Species code**	**Dataset**	**Lambda (λ)**	**Body mass (g)**	**Group size**	**Hunt Juruá**	**Hunt Uatumã**	**Activity pattern**
*Lagothrix sp*	Woolly Monkey	Lago.sp	interview	1.12	8710	19.6	1.3	0	diurnal
*Cuniculus paca*	Lowland Paca	Cuni.pa	both	1.95	9500	1	14.5	31.8	nocturnal
*Dasyprocta spp*	Agouti	Dasy.sp	both	3	4500	1.2	8.1	17.8	diurnal
*Myoprocta spp*	Acouchy	Myop.sp	camera	3	750	1	0	0	diurnal
*Echimyidae spp*	Spiny Rat	Echi.sp	camera	5	560	1	0	0	nocturnal
*Sciurus ignitus*	Bolivian Squirrel	Sciu.ig	camera	3.6	700	1.2	0	0	diurnal
*Sciurus spadiceus*	South American Red Squirrel	Sciu.sp	camera	3.5	1200	1.4	0	0	diurnal
**Birds**									
*Leptotila sp*	Dove	Lept.sp	camera	2	149	1.3	0	0	diurnal
*Mitu or Crax spp*	Currasow	Mitu.Cr	both	1.465	3000	1.6	8.3	7.9	diurnal
*Ortalis guttata*	Speckled Chachalaca	Orta.gu	camera	1.76	1200	5	0	0	diurnal
*Penelope jacquacu*	Spix's Guan	Pene.ja	camera	1.491	1280	4.9	0.3	0.5	diurnal
*Odontophorus stellata*	Wood Quail	Odon.sp	camera	1.8	310	5.4	0	0	diurnal
*Psophia spp*	Trumpeter	Psop.sp	camera	1.3	1200	5.8	0	0	diurnal
*Crypturellus spp*	Small Tinamou	Cryp.sp	both	1.9	420	1.4	2.3	0	diurnal
*Tinamus spp*	Large Tinamou	Tina.sp	both	1.5	1200	1.3	4.7	2.2	diurnal
**Reptiles**									
*Chelonoidis spp*	Red/Yellow Footed Tortoise	Chel.sp	interview	2.5	4580	1.2	0	0.8	diurnal

Hunt Juruá and Hunt Uatumã are scores of the frequency with which a given species was hunted, as determined by interviews with residents of both the Juruá and Uatumã regions.

### Camera trapping

Data acquisition took place between 2013 and 2015, between April and August to avoid the period of heaviest rainfall during which cameras are often damaged. A total of 383 camera-trap deployments (hereafter, CTD) were conducted according to a standardised deployment protocol (S1 File, Appendix D). Mean functioning camera-trap-nights (FCTN) per deployment was 31.4 ± 0.4 FCTNs. Mean nearest neighbour distance between deployments was 962.1 ± 47.4 m, although camera-traps were deployed along a ~852-km nonlinear distance along the Juruá, Uatumã and Jatapú Rivers.

Camera traps were deployed both in proximity to the peri-community agricultural mosaic and in contiguous primary *terra firme* forest along transects leading away from local communities. In the Juruá region, 132 camera-traps were deployed in the peri-community agricultural mosaic, stratified across several landscape-scale habitat types ranging from large tracts of undisturbed contiguous primary forest, to homestead areas in close proximity to community households. Due to time constraints, it was not possible to replicate the agricultural deployments in the Uatumã region.

In both the Juruá and Uatumã regions, the remaining camera trap deployments were carried out using a radial design along 6-km transects starting at an area of contiguous primary *terra firme* forest nearest the community, and radiating away from the community. Waypoints were taken at the edge of contiguous primary forest and cameras were deployed at intervals of 50m, 350m, 1000m, 3000m and 6000m Euclidean distance along the transect, which can be expressed as a near-exact log-linear scale (Pearson r = 0.983).

For each deployment, the following data were recorded: (1) identity and coordinates of the nearest local community; (2) coordinates of the camera-trap station; (3) date and time of deployment and removal; (4) in case of malfunction; date and time of last photograph; (5) habitat type; and (6) if deployed in secondary forest, age (years) since abandonment as determined by community residents.

Images were edited to improve contrast and aid species identification. Congeners from different study regions such as trumpeters (*Psophia leucoptera* and *Psophia crepitans*) were treated as a single ecospecies. Closely related species that could not be consistently identified to species level, such as *Dasypus kappleri* and *Dasypus novemcinctus*, were grouped into a single ecospecies. Images of domestic animals, humans, small passerines, primates, bats, small lizards, vultures, and insects, were excluded from further analysis. We extracted all EXIF metadata including date and time from images, and validated the deployment date and time against field notes. Images of conspecifics at any given CTD >30 min apart were defined as independent detections, which were then summed at the scale of each CTD.

The different number of FCTNs per CTD were accounted for as follows. When species detections were the response variable in statistical models, the number of FCTNs was designated as a model offset variable. Where species detections are compared graphically, or in analyses where offsets could not be used, detections were divided by the FCTNs to derive a relative abundance metric per CTD.

In the case of ambiguous images for which a subject could only be identified to a broader ecospecies, a CTD-specific detection rate was calculated for each ecospecies sub-category, and then used to apportion detections between sub-categories. If that CTD included no photographs that could be reliably identified to either sub-category, then the overall detection rate for all CTDs was used.

Species-specific camera trapping detections were multiplied by the species body mass to provide an approximate metric of individual biomass detected. Because camera traps may fail to detect some group members, we simply defined detections as a single adult of undetermined sex. In order to account for species differences in group size, this individual biomass estimate was multiplied by the mean population-scale group size to provide a measure of detected group biomass per species per CTD. Note that in this study, we do not estimate biomass per unit area, but multiply camera trap detections by average body weight and group size to provide a relative measure for comparison across camera trap sites.

Several biomass groupings and weightings were created per CDT, which summed the detected group biomass of (1) *All*—all species; (2) *Bin*.*hunt*—all species identified in interviews as hunted (see Local interviews below); (3) *Bin*.*pers*—all species identified in interviews as livestock predators, including felids, mustelids and opossums; (4) *Bin*.*huntpers*—all species either identified as hunted, or livestock predators (5) *Bin*.*unpers*—all species neither identified as hunted nor as livestock predators; (6) *Hw*—all species, weighted by the region-specific frequency with which they were hunted, derived from interviews. Lastly, in order to determine if the extremely clumped detected biomass of white-lipped peccaries had a disproportionate effect on our results, alternative groupings and weightings were created, which both excluded and included this species (S1 File, Appendixes C and G).

### Local interviews

Interviewees were local residents in their respective communities for an average of 20 ± 1.5 years. When asked how frequently they entered the forest, 54% responded “weekly”, 16% “monthly”, 13.5% “annually”, 10.1% “daily” and 5.4% “weekly/monthly”. Making the simplistic assumption that all of our 151 respondents had worked in the forest for 8 hours, once a week for the past 20 years, these interviews represent a combined total of 143 interviewee-years of experience.

Interviews were conducted in Portuguese by the authors and without the aid of translators. Interviews were recorded using a structured questionnaire and a dictaphone, and cross-validated for accuracy. Interviewees were reassured that data would be kept anonymous and confidential, and were not paid, but some had participated in paid work such as camera trapping at the time of interviews. A total of 78 interviews were conducted, with a total of 151 respondents at 59 local communities or urban neighbourhoods (hereafter, *communities*). Interview topics included encounters with forest vertebrate fauna, household-scale livelihoods, diet, hunting, and human-wildlife conflicts.

During interviews, respondents were asked to rank their livelihoods and sources of dietary protein in order of importance. They were asked to rank species by their propensity to predate livestock and their frequency of being killed by hunters (S1 File, Appendix E). Lastly, respondents were asked to estimate the time it would take, from leaving their home, to reach any given site at which a given species (or its tracks, scats and other perishable signs) could normally be encountered and the modes of transport used. Where respondents were unable to judge this, they were asked for the location of the most recent detection of that species or its perishable signs. Where a range of possible travel times were reported, an average was used, but where separate times were reported for direct encounters and encounters with signs, the lower of the two estimates was used. This partly accounted for highly elusive species, such as large felids, which respondents reported to be present, but were rarely directly encountered. In the <10% of cases where responses were given in days rather than hours, a day was assumed to be eight travel hours. We differentiated between respondents who reported with certainty that a given species had not been encountered at all in the vicinity of the community and those who said they did not know how far one would need to travel in order to encounter a given species. In the former case, we assumed that reported absences reflected a lack of detections within 24 travel hours from the community. In the latter case, we did not record an encounter distance, because we assumed that this threshold could not be confidently estimated by the respondent, perhaps through lack of experience in identifying tracks and other signs of a given species. The Manhattan distance to a species encounter was calculated using the transport time and mode of transport reported from interviews, and average transport velocity [83] (S1 File, Appendix F).

### Spatial variables

All spatial variables were extracted in ArcGIS (version 10.3). GPS waypoints were recorded at the centre of all communities, including those interviewed or in proximity to our CTDs. A transport network accounting for all main rivers, tributaries, known navigable perennial streams, roads and known tracks near all surveyed communities and CTDs was constructed from GPS track-logs taken over successive fieldwork years. We calculated the Manhattan or “transport” distance among CTDs, communities and towns across both study regions, using Network Analyst. Having identified the community/town with the shortest Manhattan distance to a given CTD/interviewed community, we then calculated the Euclidean distance between them, giving us a “hybrid” distance (see S1 File, Appendix F).

The number of households per community was determined by taking the mean values obtained from interviews conducted during this study, Projeto Médio Juruá interview archive [81] and The Sustainable Amazon Foundation (FAS) community census data [84]. The number of households per urban centre was derived from IBGE census data [85]. Hybrid distances and urban populations were combined into a single variable, the urban proximity score. This was calculated as the urban population, divided by the square-root of the hybrid distance to a given community or CTD.

For each CTD, the primary forest area (including both floodplain *várzea /igapó* forest and upland *terra firme* forest) within a 500-m buffer was calculated. Data from INPE PRODES, Global Forest Change, RADAMBRASIL, and the Instituto de Conservação e Desenvolvimento Sustentável Amazonas [86–90] were used in order to exclude deforested areas, permanent water bodies and natural non-forest vegetation, including white-sand *campina* and *campinarana*, from the 500-m buffers. *Várzea/igapó* and *terra firme* forest were not treated as separate variables because they were strongly correlated with one another and with relative elevation. PRODES and GFC datasets were cross-validated (S1 File, Appendix F).

Due to landscape-wide elevational gradients, the elevation of each CTD relative to the adjacent main river or stream was estimated using a terrain modelling method similar to that of the Height Above the Nearest Drainage (HAND), a topographic algorithm that calculates the relative elevation above the water-table [91] (see S1 File, Appendix F). A map of perennial streams was created, using data from both the IBGE “hidro tot linha” shapefile and the Hydrosheds hydrographic dataset [85,92]. The Euclidean distance between each CTD and the nearest perennial stream was then calculated.

### Statistical analysis

All statistical analyses were conducted in R (2.15.1). Collinearity between independent variables was assessed using Spearman’s rank correlation tests. Where explanatory variables had bivariate Rho >0.70, they were modelled separately. Variance inflation factors (VIFs) of the retained explanatory variables were additionally calculated in order to test for multicollinearity. Resultant VIFs below three indicated low multicollinearity [93]. Data distributions and relationships were inspected using histograms. For count data, Poisson models were attempted and where overdispersion was revealed, negative binomial models were used. For distance-to-encounter data, Gaussian (using both identity and log links) and Gamma models were tested and inspected for model fits. Variables were scaled to enable models to converge and aid variable effect size comparisons.

The ‘best’ models were selected based on their Akaike's weights (wAICc) and the ΔAICc, corrected for small sample sizes. We considered models with ΔAICc<2.0 and wAICc>0.1 as equally plausible to explain observed patterns [94]. Where multiple plausible models were retained, they were weighted and averaged using the *model*.*avg* function in the R package *MuMIn*.

To investigate the degree of depletion of our study species, negative binomial Generalised Linear Mixed-effect Models (GLMMs) were created for (a) independent detections of each species detected at >10 CTDs; (b) the total number of detections of all species per CTD; and (c) the biomass of each grouping described above. In each case, the log of the number of FCTNs was specified as an offset variable and study region and community ID were designated as nested random effects. The following anthropogenic and ecological variables as described above were included as fixed effects—the distance to the nearest community (COM.DIST), population of the nearest community (COM.POP), the urban proximity score (TOWN), the percentage of primary forest (PRIMARY), the distance to the nearest perennial stream (STREAM), and relative elevation (ELEV). An interaction between COM.DIST and COM.POP was initially specified, but it was removed as it failed to produce stronger models. Where models failed to converge, they were simplified by first removing REGION from the nested random effect, and then by removing the fixed effects with the lowest bivariate correlation with the dependent variable in question.

To investigate the impact of anthropogenic disturbance on the activity patterns of our study species, the first photograph of every independent detection per species was assigned a temporal period, with daytime specified as between 06:00h and 18:00h and night-time the converse. The number of nocturnal and diurnal detections per species, and for all species combined, were summed per CTD. Negative binomial GLMMs were created (a) for every species detected at >10 CTDs; and (b) for all species detected. GLMMs were structured as described above, except that the log of the total number of detections was specified as an offset.

In order to determine if nocturnal and cathemeral species were relatively more prevalent in areas of high anthropogenic disturbance, independent detections were categorised as either diurnal or nocturnal/cathemeral species. The number of independent detections of non-diurnal species were summed per CTD and treated as a response variable in a multivariate negative binomial GLMM, with the log of the total number of independent detections per CTD as an offset, and the community ID and region as nested random effects.

Lastly, Gamma GLMMs were used to assess the relative importance of both species traits and anthropogenic variables on the detection distances reported during interviews. Data were disaggregated such that the reported detection distance for every species from every interview was used as the dependent variable. To account for both data nestedness and phylogenetic relatedness, both species identity nested within taxonomic family (S1 File, Appendix A) and community identity, were specified as random effects. Explanatory variables included simultaneously in the initial global model were TOWN, COM.POP, the region-specific hunting score (HUNT), species intrinsic rate of increase (LAMBDA), body mass (MASS) and group size (GROUP).

## Results

### Species detections at camera trap stations

**Fig 2:**
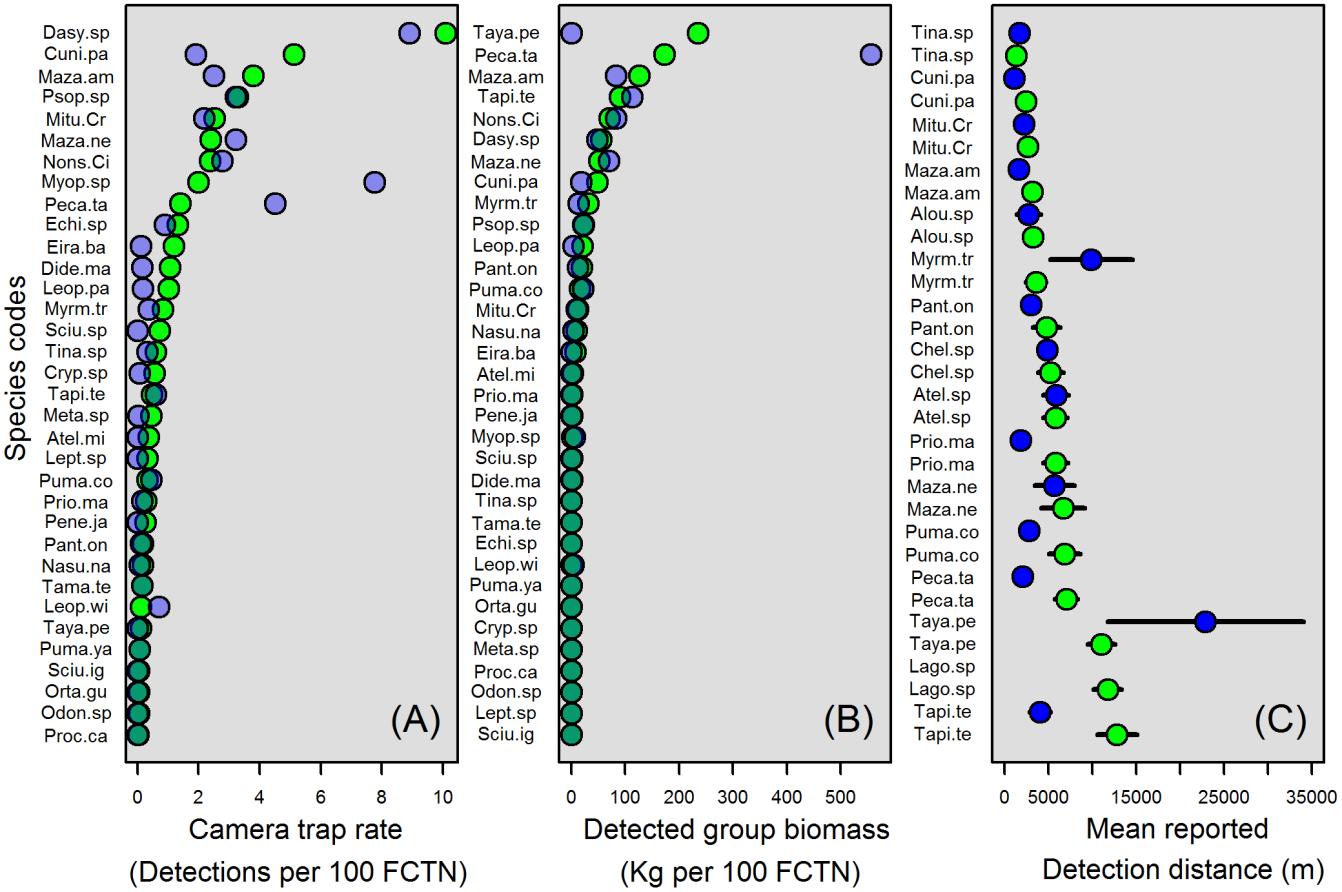
Ranked species detections from camera trap and local interview data. (A) CTR (detections per 100 FCTNs), ranked by Juruá detections from highest to lowest. (B) Group biomass (kg) detected per 100 FCTNs, ranked by Juruá detections from highest to lowest. (C) Mean interview-reported detection distance (m) and standard errors, ranked by Juruá detections, from nearest to farthest. Detections from the Juruá and Uatumã regions are indicated by green and blue circles, respectively.

A total of 38 vertebrate species (or species groups), including 29 mammals, eight birds and a forest tortoise, were either the subject of interviews, reliably detected by camera traps, or both (Table 1). A total of 34 taxa were reliably detected by camera traps (Fig 2). The 15 most frequently detected species accounted for >90% of all detections. In the Uatumã region, only 25 taxa were reliably detected. The following taxa were detected at Juruá but not at Uatumã: *Atelocynus microtis*, *Procyon cancrivorus*, *Tayassu pecari*, *Sciurus ignitus*, *Sciurus spadiceus*, *Leptotila sp*, *Odontophorus stellatus*, *Ortalis guttata* and *Penelope jacquacu*. Considering only the 10 most frequently detected species, *Cuniculus paca* was detected over twice as frequently at Juruá than at the Uatumã region, and *Myoprocta spp* was detected more than three times as frequently in the Uatumã region than at Juruá.

When species were ranked according to estimated group biomass, the 10 top-ranking species accounted for >90% of the total biomass. Between regions, the overall detected grouped biomass raking is similar except for *Tayassu pecari*, which accounted for the highest detected biomass at Juruá but was not detected at Uatumã, and *Pecari tajacu*, the biomass of which was over three times as high at Uatumã than at Juruá.

### Group biomass and species-specific models

**Fig 3:**
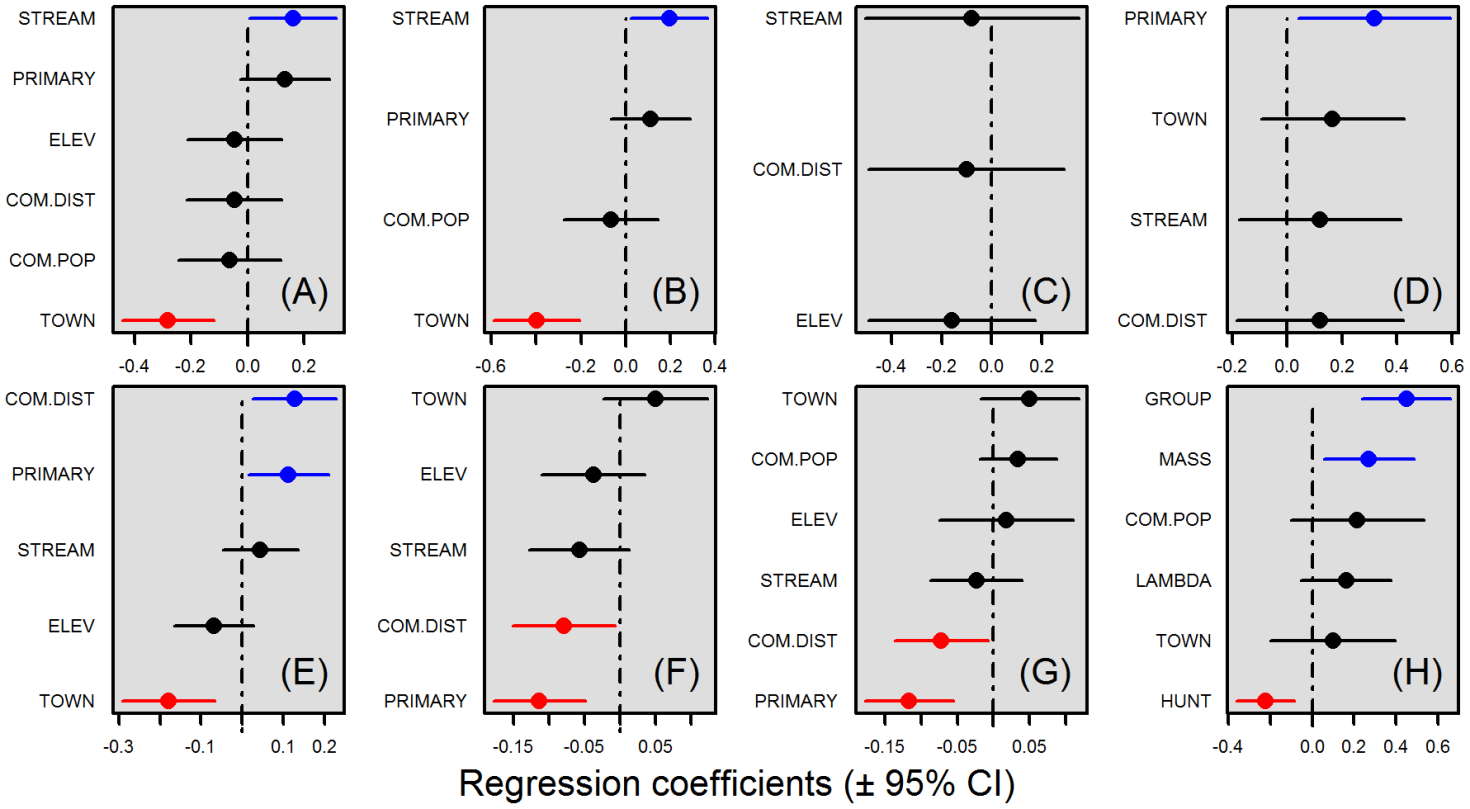
Explanatory variables retained in GLMMs examining group biomass, species activity patterns and reported detection distances. (A) Group biomass of all species. (B) Group biomass of only hunted species. (C) Group biomass of only persecuted species. (D) Group biomass of species that were neither hunted nor persecuted. (E) Detection rates of all species. (F) Degree of nocturnality of all species. (G) Proportion of nocturnal species. (H) Reported detection distance from communities of all species. Explanatory variable codes are shown to the left of each panel. Coefficients and 95% confidence intervals of the explanatory variables retained in the averaged best performing models are presented within panels. Significantly positive and negative effect sizes are coloured in blue and red, respectively.

**Fig 4:**
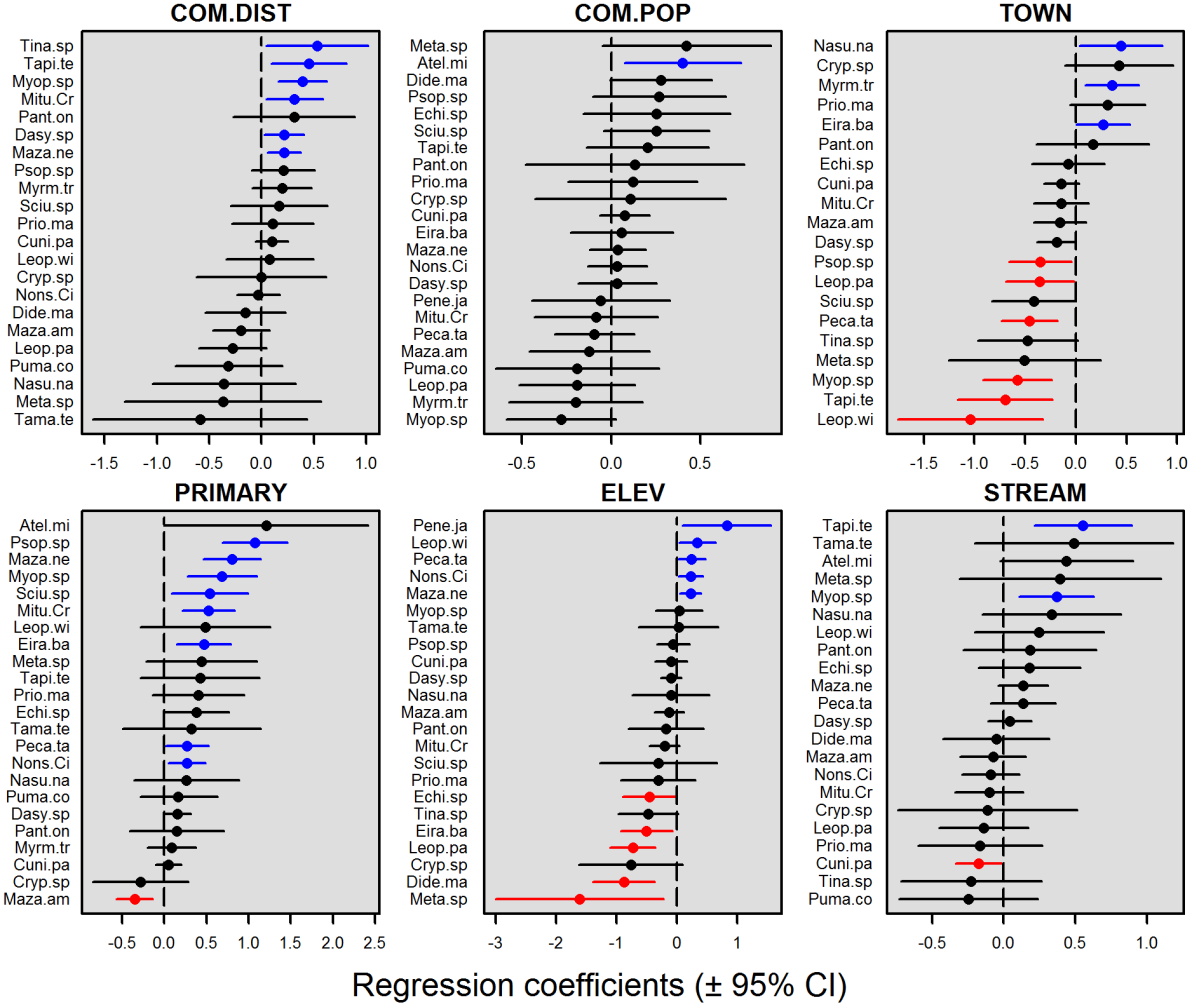
Coefficients and 95% confidence intervals of the explanatory variables retained in the averaged best performing GLMMs examining camera trap detection rates of individual species. Species codes are shown to the left of each panel, and are ranked top to bottom from the highest to the lowest coefficient. Significantly positive and negative effect sizes are coloured in blue and red, respectively.

Models of summed detections and detected group biomass were highly consistent (Fig 3 and S1 File, Appendix G). For all group-detection models that include hunted species, whether they were weighted or unweighted by hunting preference, and whether or not they included white-lipped peccary, total detections and imputed biomass were significantly depressed in proximity to urban areas. Areas close to perennial streams also had lower detected group biomass, whereas summed detections were lower in areas close to communities surrounded by low proportions of primary forest. In contrast, the biomass of species that are neither hunted nor persecuted was most strongly influenced by habitat, with a higher biomass detected in primary forest. In single-species detection models (Fig 4), no single explanatory variable significantly influenced the abundance of all species. Species responses instead appeared to be highly idiosyncratic.

### Species activity patterns

Most of our study species are diurnal (Table 1 and S1 File, Appendix B), a smaller number are nocturnal, and the minority are cathemeral. Those most often reported as hunted were a mix. Models suggest that in areas in proximity to communities with a low proportion of primary forest (a) pooled detections of the entire species assemblage were significantly more nocturnal; and (b) the proportion of nocturnal and cathemeral species detected was relatively higher (Fig 3). However, single-species activity models (S1 File, Appendix H) were weak, generally with no single variable significantly predicting the proportion of nocturnal detections, except for grey brocket deer and collared peccary. The former was detected relatively more frequently at night when close to community settlements, whilst the latter was detected more frequently at night when in areas with a low proportion of primary forest and at higher elevation.

### Local interviews

In both the Juruá and Uatumã regions, local fisheries were deemed to be a more important source of animal protein than meat from game species, and subsistence hunting was not generally deemed a preeminent livelihood activity (Table 2 and S1 File, Appendix E). Our interviews gathered detection data for a total of 16 species, one of which (woolly monkeys, *Lagothrix spp*.) does not occur in the Uatumã region. Rankings of species detection distances were comparable across regions, but tapir, giant armadillo and collared peccary were on average reportedly detected over three times farther away at the Juruá than at Uatumã, whilst giant anteater and white-lipped peccary were on average detected over twice as far away at the Uatumã than at the Juruá. In models incorporating all interview responses for all species, the variables that most strongly influenced reported detection distances were species traits, rather than anthropogenic landscape metrics such as proximity to an urban centre or size of the nearby community. The three species traits associated with encounter distances farther from settlements were large group size, large body size and low hunt frequency. Large-bodied species that form large groups were reportedly detected farthest away. Although there was a significant overlap between species from the different study regions identified as hunted, the species rankings differed (Table 1).

**Table 2:** Relative importance of different livelihood activities and food sources for local residents of the Juruá and Uatumã regions, based on semi-structured interviews.

**Important livelihoods** **(% of summed weighted scores)**	**Juruá** **(N = 50)**	**Uatumã** **(N = 24)**	**Both** **(N = 74)**
Agriculture (food crops)	43.0	55.4	46.8
Fishing	19.9	5.5	15.5
Welfare payments	14.1	8.2	12.3
NTFP extraction*	14.0	6.7	11.8
Wage labour	3.8	10.6	5.9
Subsistence hunting	3.9	0.9	3.0
Timber extraction	1.2	6.4	2.8
Livestock	0.2	6.2	2.1
**Important food-sources** **(% of responses)**	**Juruá** **(N = 49)**	**Uatumã** **(N = 23)**	**Both** **(N = 72)**
Wild fish	89.8	91.3	90.3
Forest game vertebrates	6.1	4.3	5.6
Fish and game are “equal”	4.1	4.3	4.2

* Refers to extraction of Nontimber Forest Products (NTFPs) other than vertebrate game.

## Discussion

Concerns that tropical forest reserves legally occupied by human communities are inevitably “emptied” of large-bodied terrestrial forest vertebrates, are not borne out in our study regions, which retain large game populations, despite harbouring many long-established semi-subsistence communities. Nonetheless, our camera trap and interview data support evidence that (a) landscape-scale human population density depresses the detectable aggregate biomass of the entire vertebrate assemblage; (b) nocturnal species become relatively more prevalent in areas near extractive settlements; and (c) large-group-living species, that are disproportionately affected by hunting, are virtually absent from areas in close proximity to communities. Our initial hypothesis that the detection rate of large-bodied, low-lambda species would be significantly depressed in proximity to semi-subsistence communities cannot be falsified, but as currently stated may be oversimplified.

### Models of species detections, group biomass, activity patterns and distance to encounters

In single-species models, a select few species met our expectations. The largest-bodied terrestrial mammal—the lowland tapir—was negatively impacted by both local communities and urban areas. Grey brocket deer, a shy selective browser, was strongly associated with primary forest, whereas its much larger congener (red brocket deer) was attracted to secondary growth. We did not find that species predictably fell on a gradient between low-lambda, harvest-sensitive species repulsed by communities and towns, and harvest-tolerant species that may be attracted to communities. Firstly, models were heterogeneous, incorporating both anthropogenic and environmental variables. Secondly, some smaller-bodied, high-lambda species including agoutis, acouchis and tinamous, and species typically ignored by hunters such as trumpeters, were also significantly impacted by proximity to communities and urban areas. These typically ignored small-bodied species may nonetheless be behaviourally sensitive to human disturbance and are therefore repelled by human settlements without succumbing to numerical depletion. Agoutis, acouchis and trumpeters were amongst the most frequently detected species by our camera traps and therefore even a weak repulsion signal was likely to be statistically significant. This explanation is, however, inadequate as pacas, which also exhibited high detection rates, showed no significant depletion effect, despite being highly preferred by hunters.

Grouped detection rate and biomass models paint a clear and consistent picture, suggesting that depletion is indeed occurring, but not in the isotropic, community-centric manner that we anticipated. Any biomass grouping or weighting we created that included hunted species was depressed within the wide landscape neighbourhood of urban areas, whilst group biomass of non-hunted species responded instead to habitat type. This is consistent with the positive correlation in the Médio Juruá region between catch per unit of hunting effort and distance from an urban centre, but not with local human population density [95]. This suggests that depletion is occurring primarily at a landscape rather than local scale.

Another important predictor was distance to a perennial stream. This variable captures a variety of habitat characteristics and many species including tapir are known to prefer areas near water bodies and streams [96]. Contrary to our expectations, however, overall biomass was significantly lower near streams. This is likely because proximity to perennial streams is also a proxy for accessibility to hunters. Fluvial travel is less laborious than overland travel in tropical forests, especially for hunters transporting heavy carcasses, and perennial streams are therefore important access points into hunting grounds [97]. This is especially true of both the relatively infrequent long-distance hunts carried out by community members, and the commercial hunting forays carried out by urbanites, both of which are associated with high game-extraction rates [98,65]. The stronger impact of distance to a stream and urban centres than distance to a community, suggests that hunting pressure is anisotropic and urban-centric.

Several of the vertebrate species surveyed here are known to exhibit behavioural plasticity [99]. Nonetheless, though we found that nocturnal detections were relatively more frequent in disturbed areas close to communities, weak species-specific models suggest that we cannot attribute this to species-specific shifts in activity pattern. Instead, this appears to be due to a shift in community composition towards more nocturnal and cathemeral circadian rhythms of activity. This implies that in general, species have a limited capacity to adjust to and coexist with human communities and that those species less at risk from hostile human interactions become relatively more common in disturbed areas near settlements.

Models of interview data showed that species traits were stronger predictors of distance to nearest encounters with terrestrial wildlife than were anthropogenic factors. In contrast with other studies [100], the maximum rate of increase was not found to be the most important trait. Instead we found that large-bodied, group-living species were on average detected much farther from communities than small-bodied solitary species. Reported distances suggest that in many communities, the largest-bodied and largest-group-living species were not found even within a day’s return journey on foot from the community.

### Alternative explanations

There are a number of potentially competing explanations for the dual phenomena of lower camera trapping rates in heavily-settled areas and more distant detections of larger-grouped species based on interviews. These species could be changing their behaviour, such that they become less detectable whilst still using areas near communities and urban areas. However, this explanation is implausible because (a) behavioural adaptations are unlikely to influence detection rates at unbaited camera traps placed off trails; (b) our interview respondents, who are longstanding residents fully familiar with the surroundings of their respective villages, took into account animal signs such as tracks; (c) large-grouped species, such as white-lipped peccary, are almost impossible to be overlooked, because of conspicuous bulldozing tracks, noise and scent, created by herds of hundreds of animals; and (d) we found weak evidence for species-specific shifts in activity patterns.

Alternatively, these species may be absent from forests in proximity to communities and urban areas due to baseline environmental factors, but this is also implausible. While this implies that human communities have chosen parts of the landscape that are relatively inhospitable to large-bodied forest vertebrates, the converse is likely to be the case. Human communities in the Amazon have always chosen the most favourable and productive parts of the landscape [101]. In the absence of permanent human settlements, these areas would likely have an elevated population of the large-bodied species relative to the surrounding landscape, because of the higher productivity and food availability, including fruit pulp and seeds. Lastly, these species may have been depleted in proximity to areas of high human population density, repelled from them, or both. The fact that large-bodied, large-group-living species are known to be especially vulnerable to overhunting [102,103] supports this explanation.

Species that interviewees ranked as frequently hunted were reportedly detected close to communities. This may suggest that the most hunted species are especially resilient and that hunting has little impact on the overall game assemblage. It may even suggest that hunters are deliberately or inadvertently hunting those species most able to sustain offtake. Alternatively, it may be evidence that hunters have had to switch their prey profile to smaller, more resilient species, having already depleted or repelled larger-bodied species to a distance at which it is no longer profitable to pursue them [104]. Although our study cannot discriminate between these alternative explanations, our evidence most likely supports the latter, as hunters consistently choose larger-bodied prey such as tapir and white-lipped peccaries in landscapes where they are available [105].

### Evidence for depletion

We believe that both of our study landscapes represent a “best case scenario” for subsistence hunting, which potentially underestimates the degree of faunal depletion. Firstly, our study landscapes are characterised by a high proportion of remaining primary forest and low human population density. The large tracts of primary forest beyond easy access likely act as sources, replenishing populations of terrestrial vertebrates in proximity to communities. In both regions, hunting was ranked as a less important livelihood activity and hunted meat was ranked far below fish as an important protein source; we should therefore expect the hunting pressure signal to be weak. In both regions, healthy fisheries provide for the bulk of animal protein requirements [95,106]. In the relatively wealthier Uatumã region, where regular wage labour is more common and market produce is more accessible, subsistence activities in general are less practiced [107].

Secondly, our camera trap transects are generally oriented against a primary productivity gradient. Communities and urban areas are typically located in high-productivity portions of the landscape, as commonly observed throughout human pre-history in the Amazon basin [108]. Transects leading away from communities, along which we deployed cameras, are oriented roughly perpendicular to the main river and the várzea floodplain, and are therefore along a gradient from high productivity floodplains into low-productivity upland terra firme forest. This is problematic because soil fertility mediates the density and species richness of nonvolant mammals across Amazonia [73,74]. This bias is unavoidable in our study landscapes. Deploying cameras along transects parallel to the main river would fail to achieve the goal of sampling along a hunting pressure gradient because (a) communities are situated along the river, and therefore a transect leading away from one community would simultaneously approach another; and (b) fluvial access is extremely important to hunters in our study areas, so that all camera-trap sites would be equally accessible. The fact that the imputed biomass of the entire vertebrate assemblage, as well as the camera trap detections of large, vulnerable species such as tapir, were depressed in high productivity areas close to perennial streams and communities, suggests that hunting substantially depletes wildlife.

Distance to encounter data from interviews suffers from the same problem as the farther one travels from the community into primary forest, the further one moves away from fertile floodplain. Moreover, because a high-productivity non-hunted baseline does not exist in our study regions, it is impossible to know what the abundance of different game species would be in the absence of human communities. We may assume therefore that the naturally higher productivity in proximity to communities partly masks an otherwise stronger depletion effect.

Similarly, it is possible that our snapshot study suffers from a ‘shifting baseline’ [41] if our study landscapes had already been impoverished under a post-depletion scenario, as we were unable to sample them in their faunally-intact pre-depletion state. As such, we cannot confirm whether the evidence of depletion we detected is part of a continuing trend, relative to an entirely unhunted wildlife assemblage. Though this is certainly a weakness, we doubt that either of our study regions are currently experiencing higher hunting pressure than they experienced historically. In the Uatumã region, Amazonian dark earth (locally, *terra preta)* soils indicate a long history of human habitation [109], whilst in the Juruá region, the collapse of rubber subsidies has clearly reduced the reach of hunters compared to the heyday of the Rubber Boom 50–100 years ago [62,110].

Thirdly, our camera trap data entirely neglects some of the most harvest-sensitive species, namely the large Ateline primates. Spider and woolly monkeys, which have extremely low reproductive rates compared to similarly-sized terrestrial fauna, have been shown to be some of the first species to become depleted by hunters [111,112].

Fourthly, our study does not account for the potential for hunting to reduce the group size of social vertebrate species. Our metric of detected biomass utilised a mean group size per species, which was necessary to account for large differences in group size between species, and avoid incorporating unreliable estimates of group size derived from camera trap images themselves. Nonetheless, there is some evidence that human hunting results in smaller group size for social species such as peccaries [113]. It is therefore possible that we underestimated the reduction in detected biomass in proximity to human settlements.

Lastly, our interview data can be heavily biased by detection outliers. Respondents were asked to determine the travel time to a location where one could usually encounter a given species (or its tracks or signs). Nonetheless, several respondents felt that attempting to determine a “typical” encounter location was impossible and instead recalled the nearest encounter location from recent memory. An encounter close to the community with a large forest vertebrate such as a tapir is an easily recalled event, but it may not represent a typical encounter distance. Even if tapirs are heavily depleted locally, stochastic environmental and behavioural processes dictate that rare detections close to the community will eventually occur. These outliers thus potentially lead to an underestimation of the extent of faunal depletion based on our interview data.

### Appropriate field survey methods

The above concerns thus question which field methods are most appropriate to study faunal depletion in the tropics. Line-transect methods that account only for direct animal encounters, rather than their tracks and signs, are not immune to detectability bias, because social or long-lived hunted species may alter their behaviour in hunted areas and become less detectable to observers [60]. Sign-survey methods have been proposed as a remedy, especially for terrestrial species, because such species are unable to avoid leaving tracks and signs. Sign detectability, however, varies greatly with soil substrate type and recent weather conditions [114]. Camera traps largely circumvent the aforementioned bias, as species are presumed to be unable to detect and avoid camera traps (but see [115,116]), but using photographic rates to infer relative abundance has been criticised for failing to account for detectability differences between both locations and species [117]. Our camera trap deployment approach, although not directly comparable to studies using widely adopted methodologies [118,119], was formulated to account for central-place human hunting and allow for landscape-scale replication across multiple communities.

All of the above methods are spatially and temporally limited. High financial cost and the potential to disrupt local livelihoods prohibit their intensive and long-term use in proximity to communities. Interviews avoid this problem by drawing on the long-term cognitive experience accumulated by community members with extensive knowledge of their forest environments. For instance, our camera trap dataset represents only 32.9 years of detection effort, whereas our interview data conservatively draws on roughly 4.4 times more experience. This is especially advantageous for species such as white-lipped peccary, which require extensive survey effort to accumulate sufficient independent detections on which to base meaningful inferences because of their extremely low herd densities and extremely large home range sizes. Interviews can also reduce detection biases associated with line-transect censuses as experienced hunters can quantify track and sign density. However, interview data can suffer from retrospective bias [120], social desirability bias [121], and the influence of outliers as discussed above. We therefore argue that no single technique is free from error and bias and that interview data makes a valuable contribution to understanding faunal depletion.

## Conclusions

In lowland Amazonia, conditions favourable to the persistence of game vertebrates, including modest human population densities, alternative sources of animal protein in the form of abundant fish stocks [122,123], and large areas of primary forest refugia are not uncommon [124]. Indeed, human population density in the Amazon basin is lower than in any other tropical biodiversity hotspot or wilderness area [42]. As such, the persistence of large game vertebrates in our study regions may be broadly representative of other central Amazonian sites. This does not justify complacency however. In other tropical forest regions where human population densities and game extraction rates are far higher, game depletion is likely far more severe. Moreover, human population growth rates in tropical biodiversity hotspots and wilderness areas generally exceed the average population growth rate worldwide [42]. Tropical forest management strategies, which have often proved challenging [125], may become increasingly important in human-occupied protected areas.

Despite a widely replicated study design including both camera traps and interviews, our evidence for anthropogenic depletion of terrestrial forest vertebrates is mixed. We conclude that in our “best case scenario” regions, which simultaneously retain a high proportion of primary forest cover and a low human population density with access to alternative aquatic protein, only a select few species have been depleted in proximity to communities. We found limited evidence that individual species shift their temporal activity patterns in response to human settlements. Instead, species composition is areas exposed to anthropogenic disturbance is apparently comprised of a larger fraction of nocturnal species. Interview data suggest that depletion is strongly predicated on species traits, with large-bodied large-group-living species the worst impacted. Urban areas cause landscape-scale reduction in the overall biomass of the terrestrial vertebrate assemblage. We do not know how intact the overall faunal assemblage is relative to a high-productivity, non-hunted baseline, because few exist. Strictly protected areas offer invaluable insights in this respect. We cannot further elucidate the degree to which sustainable-use protected areas effectively safeguard intact faunal assemblages, because protected status is confounded by distance to urban areas. Nonetheless, our sparsely inhabited study regions clearly retain the entire spectrum of the terrestrial vertebrate fauna, suggesting that as it stands, sustainable-use tropical forest reserves are not incompatible with biodiversity conservation.

## Supporting information

S1 File Supplementary material. Appendix A—Species taxonomic relatedness, Appendix B—Species activity patterns, Appendix C—Additional species traits, Appendix D—Camera trapping methods, Appendix E—Local interviews and livelihoods, Appendix F—Spatial data, Appendix G—Group biomass GLMMs, Appendix H—Species temporal activity pattern GLMMs, Appendix I—Protected area and study region, Appendix J—Study limitations and future directions.
(DOCX)

S2 File Interview dataset. (XLSX)

S3 File Camera trap dataset. (XLSX)
