# Supplementary material for: Measuring local depletion of terrestrial game vertebrates by central-place hunters in rural Amazonia
Source: PLoS One. 2017 Oct 17;12(10):e0186653. doi: 10.1371/journal.pone.0186653 (PMC5645145; doi:10.1371/journal.pone.0186653)
Supplement: S1 File — Appendix A—Species taxonomic relatedness, Appendix B—Species activity patterns, Appendix C—Additional species traits, Appendix D—Camera trapping methods, Appendix E—Local interviews and livelihoods, Appendix F—Spatial data, Appendix G—Group biomass GLMMs, Appendix H—Species temporal activity pattern GLMMs, Appendix I—Protected area and study region, Appendix J—Study limitations and future directions. (DOCX) [file pone.0186653.s001.docx]

**Supplementary Material**

**Measuring local depletion of terrestrial game vertebrates by central-place hunters in rural Amazonia**

Mark I. Abrahams ^1,#a*^, Carlos A. Peres ^1^, Hugo C. M. Costa ^2^

^1^ School of Environmental Sciences, University of East Anglia, Norwich, UK

^2^ PPG Ecologia e Conservação da Biodiversidade, Universidade Estadual de Santa Cruz, Ilhéus, Brazil

^#a^ Current Address: Field Conservation and Science Department, Bristol Zoological Society, Bristol, UK

* Corresponding author

Email: mabrahams@bristolzoo.org.uk (MIA)

Appendix A - Species taxonomic relatedness

Appendix B - Species activity patterns

Appendix C - Additional species traits

Appendix D - Camera trapping methods

Appendix E - Local interviews and livelihoods

Appendix F - Spatial data

Appendix G - Group biomass GLMMs

Appendix H – Species temporal activity pattern GLMMs

Appendix I - Protected area and study region

Appendix J - Study limitations and future directions

References

**Appendix A - Species taxonomic relatedness**

When creating multivariate models that included species traits, we accounted for species relatedness. Table A1 displays the taxonomic classification of each study species. Species traits models would ideally include several taxonomic levels as nested random effects as per Dulvy *et al*., 2014 [1]. Our study species are, however, spread amongst three vertebrate classes and include few congeners. We therefore included only “family” and “species” as nested random effects.

Table A1 – taxonomic classification of study species

| **Species** | **Genus** | **Family** | **Order** | **Class** |
| --- | --- | --- | --- | --- |
| *Leptotila sp* | Leptotila | Columbidae | Columbiformes | Aves |
| *Mitu or Crax spp* | Mitu/Crax | Cracidae | Galliformes | Aves |
| *Ortalis guttata* | Ortalis | Cracidae | Galliformes | Aves |
| *Penelope jacquacu* | Penelope | Cracidae | Galliformes | Aves |
| *Odontophorus stellatus* | Odontophorus | Odontophoridae | Galliformes | Aves |
| *Psophia spp* | Psophia | Psophiidae | Gruiformes | Aves |
| *Crypturellus spp* | Crypyurellus | Tinamidae | Tinamiformes | Aves |
| *Tinamus spp* | Tinamus | Tinamidae | Tinamiformes | Aves |
| *Mazama americana* | Mazama | Cervidae | Artiodactyla | Mammalia |
| *Mazama nemorivaga* | Mazama | Cervidae | Artiodactyla | Mammalia |
| *Pecari tajacu* | Pecari | Tayassuidae | Artiodactyla | Mammalia |
| *Tayassu pecari* | Tayassu | Tayassuidae | Artiodactyla | Mammalia |
| *Atelocynus microtis* | Atelocynus | Canidae | Carnivora | Mammalia |
| *Leopardus pardalis* | Leopardus | Felidae | Carnivora | Mammalia |
| *Leopardus wiedii* | Leopardus | Felidae | Carnivora | Mammalia |
| *Panthera onca* | Panthera | Felidae | Carnivora | Mammalia |
| *Puma concolor* | Puma | Felidae | Carnivora | Mammalia |
| *Puma yagouaroundi* | Puma | Felidae | Carnivora | Mammalia |
| *Eira barbara* | Eira | Mustelidae | Carnivora | Mammalia |
| *Nasua nasua* | Nasua | Procyonidae | Carnivora | Mammalia |
| *Procyon cancrivorus* | Procyon | Procyonidae | Carnivora | Mammalia |
| *Priodontes maximus* | Priodontes | Chlamyphoridae | Cingulata | Mammalia |
| *Nonspecific Cingulata small* | Unspecified.1 | Unspecified.2 | Cingulata | Mammalia |
| *Didelphis marsupialis* | Didelphis | Didelphidae | Didelphimorphia | Mammalia |
| *Metachirus spp* | Metachirus | Didelphidae | Didelphimorphia | Mammalia |
| *Tapirus terrestris* | Tapirus | Tapiridae | Perissodactyla | Mammalia |
| *Myrmecophaga tridactyla* | Myrmecophaga | Myrmecophagidae | Pilosa | Mammalia |
| *Tamandua tetradactyla* | Tamandua | Myrmecophagidae | Pilosa | Mammalia |
| *Alouatta spp* | Alouatta | Atelidae | Primates | Mammalia |
| *Ateles spp* | Ateles | Atelidae | Primates | Mammalia |
| *Lagothrix spp* | Lagothrix | Atelidae | Primates | Mammalia |
| *Cuniculus paca* | Cuniculus | Cuniculidae | Rodentia | Mammalia |
| *Dasyprocta spp* | Dasyprocta | Dasyproctidae | Rodentia | Mammalia |
| *Myoprocta spp* | Myoprocta | Dasyproctidae | Rodentia | Mammalia |
| *Echimyidae spp* | Unspecified.3 | Echimyidae | Rodentia | Mammalia |
| *Sciurus ignitus* | Sciurus | Sciuridae | Rodentia | Mammalia |
| *Sciurus spadiceus* | Sciurus | Sciuridae | Rodentia | Mammalia |
| *Chelonoidis spp* | Chelonoidis | Testudinidae | Testudines | Sauropsida |

**Appendix B – Species activity patterns**

Table A2 displays the method by which camera trap detection data per species were converted into an activity pattern designation. The first photograph of every independent detection per species was assigned a temporal period as follows (1) day – between 07:00h and 17:00h (2) night - between 19:00h and 05:00h (3) dawn/dusk - between 05:00h and 07:00h and between 17:00h and 19:00h. For each species, the proportion of detections in each category were calculated. Species with a daytime detection proportion < 0.15 were designated nocturnal. Species with a night-time detection proportion < 0.15 were designated diurnal. All other species were designated cathemeral.

Table A2 – Species activity patterns

| **Species** | **N independent camera trap detections** | **Dawn/dusk proportion** | **Day proportion** | **Night proportion** | **Activity pattern designation** |
| --- | --- | --- | --- | --- | --- |
| *Leptotila sp* | 30 | 0.13 | 0.87 | 0 | diurnal |
| *Mitu or Crax spp* | 294 | 0.21 | 0.79 | 0 | diurnal |
| *Ortalis guttata* | 5 | 0 | 1 | 0 | diurnal |
| *Penelope jacquacu* | 25 | 0.12 | 0.88 | 0 | diurnal |
| *Odontophorus stellatus* | 4 | 0.25 | 0.75 | 0 | diurnal |
| *Psophia spp* | 392 | 0.14 | 0.86 | 0 | diurnal |
| *Crypturellus spp* | 53 | 0.42 | 0.58 | 0 | diurnal |
| *Tinamus spp* | 65 | 0.4 | 0.6 | 0 | diurnal |
| *Mazama americana* | 421 | 0.22 | 0.29 | 0.49 | cathemeral |
| *Mazama nemorivaga* | 310 | 0.2 | 0.65 | 0.15 | cathemeral |
| *Pecari tajacu* | 252 | 0.1 | 0.87 | 0.04 | diurnal |
| *Tayassu pecari* | 10 | 0.2 | 0.6 | 0.2 | cathemeral |
| *Atelocynus microtis* | 34 | 0.18 | 0.79 | 0.03 | diurnal |
| *Leopardus pardalis* | 101 | 0.2 | 0.21 | 0.6 | cathemeral |
| *Leopardus wiedii* | 30 | 0.13 | 0.2 | 0.67 | cathemeral |
| *Panthera onca* | 20 | 0.2 | 0.6 | 0.2 | cathemeral |
| *Puma concolor* | 42 | 0.21 | 0.45 | 0.33 | cathemeral |
| *Puma yagouaroundi* | 8 | 0.25 | 0.75 | 0 | diurnal |
| *Eira barbara* | 114 | 0.13 | 0.85 | 0.02 | diurnal |
| *Nasua nasua* | 18 | 0.22 | 0.78 | 0 | diurnal |
| *Procyon cancrivorus* | 2 | 0 | 0 | 1 | nocturnal |
| *Priodontes maximus* | 31 | 0.23 | 0 | 0.77 | nocturnal |
| *Nonspecific Cingulata small* | 297 | 0.03 | 0 | 0.97 | nocturnal |
| *Didelphis marsupialis* | 103 | 0.12 | 0 | 0.88 | nocturnal |
| *Metachirus spp* | 43 | 0.14 | 0 | 0.86 | nocturnal |
| *Tapirus terrestris* | 60 | 0.22 | 0.05 | 0.73 | nocturnal |
| *Myrmecophaga tridactyla* | 87 | 0.23 | 0.68 | 0.09 | diurnal |
| *Tamandua tetradactyla* | 18 | 0.11 | 0.11 | 0.78 | nocturnal |
| *Cuniculus paca* | 530 | 0.05 | 0 | 0.95 | nocturnal |
| *Dasyprocta spp* | 1180 | 0.3 | 0.7 | 0.01 | diurnal |
| *Myoprocta spp* | 396 | 0.62 | 0.37 | 0.01 | diurnal |
| *Echimyidae spp* | 146 | 0.17 | 0 | 0.83 | nocturnal |
| *Sciurus ignitus* | 5 | 0 | 1 | 0 | diurnal |
| *Sciurus spadiceus* | 68 | 0.03 | 0.97 | 0 | diurnal |

**Appendix C - Additional species traits**

In the Juruá region, an additional 107 interviews, which focused in greater detail on semi-subsistence farming and crop raiding, were conducted at 24 communities. Terrestrial vertebrate species were assigned a rank-weighted agricultural score based on their propensity to enter agricultural plots [2]. On the basis of Wilman *et al*., 2014 [3] and C.A. Peres (unpubl. data), species were also assigned an ordinal trophic score, with lower numbers indicating species feeding at a lower trophic level. The trophic and agricultural scores (Table A3) were initially considered as explanatory trait variables in GLMMs of interview-reported detection distance. The agricultural score was however excluded because it co-varied with the hunting score. The trophic score was subsequently excluded (as was the species pattern designation) to allow models to converge. Lastly, a biomass grouping, which summed the detected group biomass of all species, weighted by their propensity to enter agricultural plots, was created (biomass grouping code RW). This was modelled in the same way as the other biomass groupings, as described in the statistical analysis section of the manuscript.

Table A3 – Species agricultural and trophic scores

| **Species** | **Agricultural score** | **Trophic score** |
| --- | --- | --- |
| *Leptotila sp* | 1.7 | 3 |
| *Mitu or Crax spp* | 2.3 | 3 |
| *Ortalis guttata* | 0.5 | 3 |
| *Penelope jacquacu* | 0.1 | 3 |
| *Odontophorus stellatus* | 0 | 3 |
| *Psophia spp* | 0 | 4 |
| *Crypturellus spp* | 1.2 | 3 |
| *Tinamus spp* | 1.2 | 3 |
| *Mazama americana* | 19.4 | 2 |
| *Mazama nemorivaga* | 0 | 2 |
| *Pecari tajacu* | 15.9 | 4 |
| *Tayassu pecari* | 0.2 | 4 |
| *Atelocynus microtis* | 0 | 5.5 |
| *Leopardus pardalis* | 0.1 | 6 |
| *Leopardus wiedii* | 0.5 | 6 |
| *Panthera onca* | 1.7 | 6 |
| *Puma concolor* | 1.2 | 6 |
| *Puma yagouaroundi* | 0 | 6 |
| *Eira barbara* | 1.4 | 5.5 |
| *Nasua nasua* | 0 | 5.5 |
| *Procyon cancrivorus* | 0 | 5.5 |
| *Priodontes maximus* | 1.6 | 5 |
| *Nonspecific Cingulata small* | 9.3 | 5 |
| *Didelphis marsupialis* | 0 | 4 |
| *Metachirus spp* | 0 | 4 |
| *Tapirus terrestris* | 3.1 | 2 |
| *Myrmecophaga tridactyla* | 0.5 | 5 |
| *Tamandua tetradactyla* | 0 | 4.5 |
| *Alouatta spp* | 0 | 2 |
| *Ateles spp* | 0 | 3 |
| *Lagothrix spp* | 0 | 3 |
| *Cuniculus paca* | 13.3 | 3 |
| *Dasyprocta spp* | 23.3 | 3 |
| *Myoprocta spp* | 0.1 | 3 |
| *Echimyidae spp* | 0.8 | 3 |
| *Sciurus ignitus* | 0 | 3 |
| *Sciurus spadiceus* | 0 | 3 |
| *Chelonoidis spp* | 0 | 2 |

**Appendix D - Camera trapping methods**

Bushnell Trophy-Cam and Reconyx HC500 Hyperfire camera trap models were used. Camera sensors were set to high sensitivity. They took 3 and 5 sequential burst photographs or 10- second video clip. A red light flash was used in low light conditions in order to avoid startling the subject. Cameras were deployed at knee height, attached to trees. Thin vegetation was cleared in a cone of 7 paces long by 7 paces wide in-front of the camera traps to permit detection and avoid detecting the movement of vegetation. Walk-tests were performed to confirm correct positioning. Mothballs and tampons were placed inside cameras along with batteries to repel insects and protect against humidity. A GPS waypoint was always taken. Deployment locations were not chosen to deliberately maximise detection (for example underneath fruiting trees), but conspicuous obstacles to detection were systematically avoided. All camera traps were unbaited. Deployment locations were chosen to have relatively flat ground, relatively unobscured by large trees and other obstacles. Camera traps were not deployed in seasonally flooded *várzea* forests. In order to control for the bias introduced by species-specific responses to trails, deployments along transects in primary forest were always deployed ~20m away from trails and facing away from the trail where possible. These transects made use of Projeto Médio Juruá (http://www.projetomediojurua.org/) or Programa de Monitoramento da Biodiversidade e do Uso de Recursos Naturais (ProBUC) faunal monitoring trails where available. Where such trails were unavailable, suitable transects were identified from basemaps in a GIS to avoid impassable rivers and backwater swamps and temporary trails were cut by ourselves. In areas where unforeseen obstacles such as swamps made deployment at the designated distance along the transect impossible, or if it was judged to be too late in the day to reach 6km and return before nightfall, cameras were deployed in the closest available location. Deployments outside contiguous primary forest were at least 25m from the habitat edge to control for immediate edge effects. When deploying cameras in proximity to local communities, a community resident was employed to aid in the identification of suitable locations and determine the age of secondary growth (locally *capoeiras)* since abandonment. The bulk of our camera-trap deployments were in contiguous primary forest. It was therefore not possible to investigate habitat effects in detail. Instead, we measured and analysed the proportion of primary forest within the neighbourhood of each camera. We attempted to deploy all cameras for 30 days, but malfunction and theft occasionally resulted in inconsistent deployment durations. When cameras were removed, a note was made of any problems or malfunctions such as water ingress, insect attack, dislodgement or battery failure.

Camera trap images were separated into deployment subfolders corresponding to species and species functional groups. We extracted all EXIF metadata including date and time from subfolders using the Picture Information Extractor software [4]. Data were compared with field notes and date/time were corrected where necessary.

**Appendix E - Local interviews and livelihoods**

When summarising interview data, where single numeric responses were given, means were calculated. Where multiple unranked categorical responses were given, the number of responses per category were summed. Where multiple ranked categorical responses were given, a summed, rank-weighted score per category was calculated.

In our study regions, semi-subsistence agriculture consisted mainly of *Manihot esculenta* (manioc), but also included *Musa acuminate* and *Musa balbisiana* (banana), *Zea mays* (maize), *Cucurbita* (squash), *Citrullus lanatus* var. *lanatus* (watermelon) and *Ananas comosus* (pineapple). Fishing was practiced both for subsistence and as part of a fisheries management program. Social welfare programs included the Bolsa Familia, Bolsa Verde and Bolsa Floresta. Extractive activities included rubber-tapping and other nontimber resource harvesting, including *Euterpe preccatoria* (*açaí*), *Astrocaryum murumuru* (*murumuru*), *Carapa guianensis* (*andiroba*), *Copaifera multijuga* oleoresin (*copaiba*), and *Heteropsis flexuosa* (*cipó titica*). Hunting was practiced for subsistence only. Wage labour included agricultural day-labouring as well as teaching and administrative positions. Timber-based livelihoods included timber harvesting and processing, sale of logs and sawn boards, and carpentry. Small-scale husbandry of livestock included cattle, pigs, chickens and goats.

**Appendix F - Spatial data**

To convert reported travel times into transport distances, we applied average speeds per transport type following Parry and Peres, 2015 [5] (i.e. motorised canoe: 9km/h, un-motorised canoe: 5km/h, on foot: 4km/h). These mean speeds were validated by our own fieldwork experience. When calculating distances between CTDs, communities and towns, to be used in GLMMs of camera trap data, raw Euclidean distances were deemed inadequate because they do not account for the physical barriers and enablers that influence human travel costs across the landscape. Likewise, raw transport/Manhattan distance was not used, because forest vertebrates using the landscape are not hindered or enabled by features such as rivers in the same way as humans. Instead an intermediate “hybrid” measure was preferred, as described in the methods section of the manuscript.

Deforestation and human population GIS datasets were selected and validated as follows. We compared the area of available data and degree of cloud cover associated with the annual INPE PRODES raster landcover datasets between 2007 and 2013 [6]. The 2009 dataset was chosen for further analysis, because it has the largest area of data, the smallest area of cloud and corresponds closely in time to the 2007-2009 IBGE population census.

The aforementioned PRODES dataset was compared to the Hansen et al. (2013) Global Forest Change (GFC) dataset [7]. The GFC “Year of gross forest cover loss event” raster layer was used to compare forest cover losses that occurred before 2009. For both datasets, cells were reclassified into either deforested or not deforested. For GFC data, we defined any pixel >50% deforested as “deforested”. A subset of data for which there was both PRODES and GFC data was taken from the 00N_070W degree granule in the state of Amazonas. The data were aligned and aggregated to a cell size of ~12500m, representing a 100 cell factor aggregation of the PRODES dataset. A 30-km buffer was erased from this area to exclude edge effects, leaving an area of analysis of 1,072,620 km².

Additionally, a spatially explicit dataset from the 2007-2009 IBGE population census of rural households in the Brazilian states of Amazonas, Pará, Acre, Mato Grosso, Rondônia and Roraima (each point representing one permanent, private, rural household), was aggregated at the level of census sector and municipality, and compared to publicly available IBGE 2007 census data [8].

For both deforestation and human population datasets, Spearman’s rank correlation tests were performed. The human population datasets were strongly correlated, whether aggregated by census sector or by municipality (N = 8314, Spearman’s Rho = 0.69 and N= 721, Spearman’s Rho = 0.85, respectively). Likewise, the deforestation datasets were strongly correlated (N = 6854. Spearman’s Rho = 0.71).

Although the GFC data is of a higher resolution (30m rather than 120m pixels) and extends to 2014, it does not account for deforestation that occurred before the year 2000. The PRODES and GFC datasets were therefore combined into a single deforestation polygon. Cells were reclassified into either deforested or not deforested. Deforested pixels were converted to polygons and merged, such that any areas defined as deforested in either dataset were ultimately defined as deforested. Unlike the PRODES data, the GFC data: (1) is of a 30-m pixel resolution, (2) corrects for cloud cover, (3) classifies pixels by their percentage deforestation, (4) defines any vegetation above 5m in height as forest and, and (5) does not classify natural non-forest vegetation.

When considering the impact on forest vertebrates of terrain elevation above the level of the local drainage, elevation above mean sea level is inappropriate due to landscape-wide elevational gradients. There are 24m and 22m elevational differences between the extremes of our study landscapes along the Juruá and Uatumã River drainages, respectively, and a roughly 65m elevational difference between study regions. Therefore, the elevation of each CTD relative to that of the adjacent main river or stream was calculated as follows. First, the elevation of each CTD was extracted from the Shuttle Radar Topography Mission (SRTM) 30-m resolution elevation data [9]. To then calculate the elevation of the river in proximity to each deployment, a point shapefile of camera deployments was snapped to a polyline of the main rivers in our study regions. A 500-m buffer around each snapped point was then created and the lowest elevation within the buffer was extracted. The buffer ensured that the true elevation of the river would be captured, rather than that of the nearby banks and levees. The relative elevation per CTD was then taken to be the elevational difference between the CTD itself and the corresponding point on the main river.

**Appendix G – Group biomass GLMMs**

Table A4 - Explanatory variables retained in GLMMs of detected group biomass, along with coefficients and 95% confidence intervals. Biomass groupings that end “.nq” are those which exclude white-lipped peccaries.

| **Biomass grouping** | **Explanatory variable** | **Coefficient** | **Lower CI** | **Upper CI** |
| --- | --- | --- | --- | --- |
| All | COM.DIST | -0.04657 | -0.215793366 | 0.1226479 |
| All | COM.POP | -0.063 | -0.245696803 | 0.1196944 |
| All | ELEV | -0.04628 | -0.21250274 | 0.1199453 |
| All | PRIMARY | 0.13324 | -0.026023411 | 0.2925126 |
| All | STREAM | 0.16131 | 0.007864529 | 0.3147489 |
| All | TOWN | -0.28228 | -0.445864681 | -0.1186953 |
| All.nq | COM.DIST | 0.09012 | -0.04692327 | 0.2271641 |
| All.nq | ELEV | 0.0291 | -0.10637828 | 0.1645801 |
| All.nq | PRIMARY | 0.07321 | -0.072167 | 0.2185914 |
| All.nq | STREAM | 0.15184 | 0.02147715 | 0.282208 |
| All.nq | TOWN | -0.27241 | -0.40240011 | -0.1424228 |
| Bin.hunt | COM.POP | -0.06411 | -0.27534529 | 0.1471325 |
| Bin.hunt | PRIMARY | 0.11254 | -0.06553513 | 0.2906117 |
| Bin.hunt | STREAM | 0.1959 | 0.02430619 | 0.3674946 |
| Bin.hunt | TOWN | -0.39891 | -0.59152461 | -0.2062854 |
| Bin.hunt.nq | COM.DIST | 0.10934 | -0.04829083 | 0.2669728 |
| Bin.hunt.nq | ELEV | 0.03639 | -0.12347509 | 0.196256 |
| Bin.hunt.nq | PRIMARY | 0.06741 | -0.09291359 | 0.2277377 |
| Bin.hunt.nq | STREAM | 0.18885 | 0.04150766 | 0.3361958 |
| Bin.hunt.nq | TOWN | -0.38499 | -0.54377005 | -0.2262146 |
| Bin.huntpers | COM.POP | -0.06461 | -0.25800878 | 0.1287847 |
| Bin.huntpers | ELEV | -0.02542 | -0.20579627 | 0.1549517 |
| Bin.huntpers | PRIMARY | 0.12514 | -0.05117085 | 0.3014532 |
| Bin.huntpers | STREAM | 0.17802 | 0.01220641 | 0.3438268 |
| Bin.huntpers | TOWN | -0.34798 | -0.53109371 | -0.1648709 |
| Bin.huntpers.nq | COM.DIST | 0.0937 | -0.05829882 | 0.2457058 |
| Bin.huntpers.nq | PRIMARY | 0.05955 | -0.09653755 | 0.215643 |
| Bin.huntpers.nq | STREAM | 0.16717 | 0.02430702 | 0.3100339 |
| Bin.huntpers.nq | TOWN | -0.34023 | -0.49005394 | -0.1904038 |
| Bin.pers | COM.DIST | -0.1003 | -0.491194 | 0.2905908 |
| Bin.pers | ELEV | -0.1592 | -0.4929438 | 0.1746407 |
| Bin.pers | STREAM | -0.0792 | -0.5069253 | 0.3485156 |
| Bin.unpers | COM.DIST | 0.1192 | -0.1849999 | 0.4233265 |
| Bin.unpers | PRIMARY | 0.3169 | 0.0399003 | 0.5939337 |
| Bin.unpers | STREAM | 0.1194 | -0.175805 | 0.4146678 |
| Bin.unpers | TOWN | 0.1657 | -0.093652 | 0.425036 |
| Hw | COM.POP | -0.07841 | -0.29966065 | 0.142834 |
| Hw | PRIMARY | 0.14465 | -0.04338659 | 0.3326929 |
| Hw | STREAM | 0.17915 | -0.00026673 | 0.3585703 |
| Hw | TOWN | -0.40415 | -0.60842744 | -0.1998696 |
|  |  |  |  |  |
| Hw.nq | ELEV | 0.05071 | -0.11700378 | 0.2184301 |
| Hw.nq | PRIMARY | 0.07152 | -0.09270986 | 0.2357516 |
| Hw.nq | STREAM | 0.177 | 0.02466062 | 0.3293347 |
| Hw.nq | TOWN | -0.40673 | -0.56990851 | -0.2435509 |
|  |  |  |  |  |
|  |  |  |  |  |
|  |  |  |  |  |
|  |  |  |  |  |
|  |  |  |  |  |
|  |  |  |  |  |
|  |  |  |  |  |
|  |  |  |  |  |
|  |  |  |  |  |
|  |  |  |  |  |

**Appendix H – Species temporal activity pattern GLMMs**

Table A5 - Explanatory variables retained in GLMMs of activity patterns per species, along with coefficients and 95% confidence intervals.

| **Species code** | **Explanatory variable** | **Coefficient** | **Lower CI** | **Upper CI** |
| --- | --- | --- | --- | --- |
| Atel.mi | COM.POP | 0.5305 | -0.137108 | 1.1981521 |
| Cryp.sp | COM.POP | -2.0551 | -6.800975 | 2.690798 |
| Dasy.sp | COM.DIST | -0.09429 | -0.3359992 | 0.14741811 |
| Dasy.sp | ELEV | 0.06982 | -0.170553 | 0.31018618 |
| Dasy.sp | PRIMARY | 0.04156 | -0.1928192 | 0.27594678 |
| Dasy.sp | STREAM | -0.23526 | -0.498507 | 0.02797773 |
| Dasy.sp | TOWN | 0.07292 | -0.2973185 | 0.44316789 |
| Eira.ba | COM.DIST | -0.9346 | -2.616083 | 0.7468972 |
| Eira.ba | PRIMARY | -0.4271 | -1.179803 | 0.3256797 |
| Leop.pa | COM.DIST | -0.11857 | -0.4888043 | 0.2516564 |
| Leop.pa | COM.POP | 0.12805 | -0.1706098 | 0.4267178 |
| Leop.pa | PRIMARY | -0.06726 | -0.2519796 | 0.1174509 |
| Maza.am | COM.DIST | -0.1764 | -0.40588004 | 0.05308596 |
| Maza.am | COM.POP | -0.11329 | -0.34842134 | 0.12184729 |
| Maza.am | ELEV | 0.06759 | -0.07706922 | 0.21224712 |
| Maza.am | PRIMARY | -0.08832 | -0.20957021 | 0.0329231 |
| Maza.am | STREAM | 0.15869 | -0.03402475 | 0.35139911 |
| Maza.ne | COM.DIST | -0.3832 | -0.67417625 | -0.0922307 |
| Maza.ne | COM.POP | 0.13039 | -0.06509588 | 0.32588558 |
| Maza.ne | ELEV | 0.09613 | -0.11636557 | 0.30862441 |
| Maza.ne | PRIMARY | 0.18358 | -0.32961615 | 0.69677907 |
| Maza.ne | STREAM | -0.20929 | -0.50163159 | 0.08304189 |
| Maza.ne | TOWN | -0.10492 | -0.37948247 | 0.16963776 |
| Mitu.Cr | COM.DIST | -0.1339 | -0.72352017 | 0.4557991 |
| Mitu.Cr | COM.POP | 0.239 | -0.22008429 | 0.6980296 |
| Mitu.Cr | ELEV | -0.2313 | -0.83982728 | 0.3771631 |
| Mitu.Cr | PRIMARY | -0.4186 | -1.03010367 | 0.1928591 |
| Mitu.Cr | STREAM | 0.4964 | -0.02822918 | 1.0209994 |
| Myop.sp | COM.DIST | 0.08608 | -0.1305849 | 0.3027441 |
| Myop.sp | COM.POP | -0.1447 | -0.62537 | 0.3359673 |
| Myop.sp | PRIMARY | -0.24435 | -0.8089356 | 0.3202404 |
| Myop.sp | TOWN | -0.17719 | -0.5055317 | 0.1511595 |
| Myrm.tr | ELEV | -0.4863 | -1.345023 | 0.3723529 |
| Nasu.na | ELEV | -4.2551 | -13.283299 | 4.773062 |
| Nasu.na | STREAM | 0.8242 | -4.419474 | 6.06782 |
| Pant.on | PRIMARY | -0.5894 | -1.42031909 | 0.2414797 |
| Pant.on | TOWN | 0.6514 | -0.08919433 | 1.392084 |
| Peca.ta | ELEV | 0.5241 | 0.004017344 | 1.044277 |
| Peca.ta | PRIMARY | -0.7368 | -1.359364566 | -0.114298 |
| Peca.ta | STREAM | 0.3858 | -0.227706078 | 0.9993084 |
| Peca.ta | TOWN | 0.5274 | -0.172840565 | 1.2276039 |
| Psop.sp | COM.DIST | -0.03811 | -0.730574 | 0.654353 |
| Psop.sp | COM.POP | -1.28468 | -3.4088527 | 0.839488 |
| Psop.sp | ELEV | -0.15088 | -0.8029862 | 0.5012211 |
| Psop.sp | PRIMARY | -0.59281 | -1.769722 | 0.5840935 |
| Psop.sp | STREAM | 0.32448 | -0.2335126 | 0.8824752 |
| Psop.sp | TOWN | -0.0289 | -0.7663993 | 0.7085985 |
| Sciu.sp | COM.POP | -1.418 | -403.7307 | 400.8953 |
| Sciu.sp | ELEV | -2.815 | -311.1659 | 305.5369 |
| Tina.sp | STREAM | -0.7874 | -2.293957 | 0.7190751 |

**Appendix I – Protected area and study region**

Wilcoxon rank sum tests were performed to determine if CTDs and surveyed communities in different study regions and in locations falling under different legally protected status, also differed with respect to our other explanatory variables. Wilcoxon tests showed that camera-trap stations and interviewed communities inside protected areas had significantly lower urban proximity scores than those outside of protected areas (p < 0.01 and < 0.001 respectively). Further Wilcoxon tests also showed that community size, urban proximity score, proportion of primary forest, distance to stream, and elevation all differed significantly by region (p <0.05, < 0.001, <0.05, <0.01, and < 0.001, respectively). Protected status and study region were therefore not included as explanatory variables in multivariate models. Region was however included as a nested random effect in negative binomial GLMMs.

To separately test the effects of protection and study region on detection rates, the number of detections of every species at every camera was modelled as the dependent variable in a negative binomial GLMM with sampling effort (FCTNs) as an offset variable, protected status or region as explanatory variables, and both species and camera IDs as random effects. To test for the effect of protection on overall biomass, the detected group biomass for all species, summed per camera, was modelled as the dependent variable in a negative binomial GLMM with sampling effort as an offset, protected status or region as explanatory variables, and camera ID as a random effect. To further test the effect of protection and study region on depletion envelopes around communities, detection distances of each species reported from each interview was modelled as the dependent variable in a Gamma GLMM with protected status or region as explanatory variables, and both species and community ID as random effects.

Camera trap detection rates inside protected areas were significantly higher and associated with a higher overall group biomass than those outside protected areas (p < 0.05 in both cases). However, respondents outside protected areas did not necessarily report significantly longer overall encounter distances than respondents inside protected areas (p = 0.06). Study region had no significant effect on overall detections, detected biomass or encounter distance (p > 0.05 in all cases)

**Appendix J – Study limitations and future directions**

Our study design could have been strengthened in a number of ways. We lacked additional camera trap sampling effort in areas of very high hunting pressure. We believe that in our study landscapes, where hunting is infrequent, a strong depletion signal is only apparent relatively close to communities. Had time permitted, we would therefore have sampled the peri-community agricultural mosaics in the Uatumã region, and devoted more sampling effort to areas near towns in both landscapes. This strategy has drawbacks, however, in that areas close to communities were more deforested, thereby adding a challenge in disentangling these effects. One could sample primary forest remnants in proximity to communities to partly mitigate this. Another fruitful approach would be to sample multiple landscapes, in which the availability of aquatic protein resources, and therefore the necessity to hunt, varies greatly. In order to avoid the confounding effects of productivity gradients, one would need to sample highly productive, but unhunted areas in tandem with adjacent hunted areas [10]. Such experimentally ideal landscapes are rare or non-existent, however, partly due to the high opportunity cost of setting them aside as strictly protected areas. Alternatively, one could conduct longitudinal studies [11], ideally by either investigating areas before and after colonisation, or during the process of abandonment. Such studies, although valuable, are again rare due to the difficulty of establishing and maintaining a long-term research presence in rural tropical areas. Although we investigated faunal depletion, investigating the sustainability of subsistence hunting in these regions was beyond the scope of this study. To do so would require both long-term and detailed hunting offtake data in tandem with species abundance data.

**References**

1. Dulvy NK, Fowler SL, Musick JA, Cavanagh RD, Kyne PM, Harrison LR, Carlson JK, Davidson LN, Fordham SV, Francis MP, Pollock CM. Extinction risk and conservation of the world’s sharks and rays. Elife. 2014 Jan 21;3:e00590.
2. Abrahams MI, Peres CA, Costa HCM. Farmers perceptions of agricultural losses caused by terrestrial forest vertebrates in Amazonia: implications for conserving biodiversity. Journal of Wildlife Management; in revision.
3. Wilman H, Belmaker J, Simpson J, de la Rosa C, Rivadeneira MM, Jetz W. EltonTraits 1.0: Species‐level foraging attributes of the world's birds and mammals. Ecology. 2014 Jul 1;95(7):2027-.
4. Picmeta Systems, [Computer software]. 2016. Picture Information Extractor. Retrieved from http://www.picmeta.com/products/picture-information-extractor.htm
5. Parry L, Peres C. Evaluating the use of local ecological knowledge to monitor hunted tropical forest wildlife over large spatial scales. Ecology and Society. 2015;20(3).
6. Instituto Nacional de Pesquisas Espaciais [INPE], 2009, Monitoramento da Floresta Amazônica Brasileira por Satélite [PRODES], http://www.obt.inpe.br/prodes/index.php, accessed 01/10/2015
7. Hansen MC, Potapov PV, Moore R, Hancher M, Turubanova SA, Tyukavina A, Thau D, Stehman SV, Goetz SJ, Loveland TR, Kommareddy A. High-resolution global maps of 21st-century forest cover change. Science. 2013 Nov 15;342(6160):850-3.
8. Instituto Brasileiro de Geografia e Estatística [IBGE], 2007. Censo Demografico, http://ibge.gov.br , accessed 01/10/2015
9. Jarvis, A., Reuter, H.I., Nelson, A., Guevara, E. 2008. Hole-filled seamless SRTM data V4, International Centre for Tropical Agriculture (CIAT). URL http:// srtm.csi.cgiar.org. Accessed: 01/10/2015
10. Arcese P, Sinclair AR. The role of protected areas as ecological baselines. Journal of Wildlife Management. 1997 Jul 1:587-602.
11. Hill K, McMillan G, Fariña R. Hunting‐Related Changes in Game Encounter Rates from 1994 to 2001 in the Mbaracayu Reserve, Paraguay. Conservation biology. 2003 Oct 1;17(5):1312-23.
